# Summoning, No-Signalling and Relativistic Bit Commitments

**DOI:** 10.3390/e21050534

**Published:** 2019-05-25

**Authors:** Adrian Kent

**Affiliations:** 1 Centre for Quantum Information and Foundations, DAMTP, Centre for Mathematical Sciences, University of Cambridge, Wilberforce Road, Cambridge CB3 0WA, UK; apak@damtp.cam.ac.uk; 2 Perimeter Institute for Theoretical Physics, 31 Caroline Street North, Waterloo, ON N2L 2Y5, Canada

**Keywords:** relativistic quantum information, quantum cryptography, summoning, no-cloning, no-signalling, bit commitment

## Abstract

Summoning is a task between two parties, Alice and Bob, with distributed networks of agents in space-time. Bob gives Alice a random quantum state, known to him but not her, at some point. She is required to return the state at some later point, belonging to a subset defined by communications received from Bob at other points. Many results about summoning, including the impossibility of unrestricted summoning tasks and the necessary conditions for specific types of summoning tasks to be possible, follow directly from the quantum no-cloning theorem and the relativistic no-superluminal-signalling principle. The impossibility of cloning devices can be derived from the impossibility of superluminal signalling and the projection postulate, together with assumptions about the devices’ location-independent functioning. In this qualified sense, known summoning results follow from the causal structure of space-time and the properties of quantum measurements. Bounds on the fidelity of approximate cloning can be similarly derived. Bit commitment protocols and other cryptographic protocols based on the no-summoning theorem can thus be proven secure against some classes of post-quantum but non-signalling adversaries.

## 1. Introduction

To define a summoning task [1,2], we consider two parties, Alice and Bob, who each have networks of collaborating agents occupying non-overlapping secure sites throughout space-time. At some point *P*, Bob’s local agent gives Alice’s local agent a state $|\psi \rangle$. The physical form of $|\psi \rangle$ and the dimension of its Hilbert space *H* are pre-agreed; Bob knows a classical description of $|\psi \rangle$ but from Alice’s perspective it is a random state drawn from the uniform distribution on *H*. At further pre-agreed points (which are often taken to all be in the causal future of *P*, though this is not necessary), Bob’s agents send classical communications in pre-agreed form, satisfying pre-agreed constraints, to Alice’s local agents, which collectively determine a set of one or more valid return points. Alice may manipulate and propagate the state as she wishes but must return it to Bob at one of the valid return points. We say a given summoning task is *possible* if there is some algorithm that allows Alice to ensure that the state is returned to a valid return point for any valid set of communications received from Bob.

The “no-summoning theorem” [1] states that summoning tasks in Minkowski space are not always possible. We write Q≻P if the space-time point *Q* is in the causal future of the point *P* and Q⊁P otherwise; we write Q⪰P if either Q≻P or Q=P and Q⋡P otherwise. Now, for example, consider a task in which Bob may request at one of two “call” points $c_{i}≻P$ that the state be returned at a corresponding return point $r_{i}≻c_{i}$, where $r_{2}⋡c_{1}$ and $r_{1}⋡c_{2}$. An algorithm that guarantees that Alice will return the state at $r_{1}$ if it is called at $c_{1}$ must work independently of whether a call is also made at $c_{2}$, since no information can propagate from $c_{2}$ to $r_{1}$; similarly if 1 and 2 are exchanged. If calls were made at both $c_{1}$ and $c_{2}$, such an algorithm would thus generate two copies of $|\psi \rangle$ at the space-like separated points $r_{1}$ and $r_{2}$, violating the no-cloning theorem. This distinguishes relativistic quantum theory from both relativistic classical mechanics and non-relativistic quantum mechanics, in which summoning tasks are always possible provided that any valid return point is in the (causal) future of the start point *P*.

Further evidence for seeing summoning tasks as characterising fundamental features of relativistic quantum theory was given by Hayden and May [3], who considered tasks in which a request is made at precisely one from a pre-agreed set of call points $\left\{c_{1},\ldots ,c_{n}\right\}$; a request at $c_{i}$ requires the state to be produced at the corresponding return point $r_{i}≻c_{i}$. They showed that, if the start point *P* is in the causal past of all the call points, then the task is possible if and only if no two causal diamonds $D_{i}=\left\{x:r_{i}⪰x⪰c_{i}\right\}$ are spacelike separated. That is, the task is possible unless the no-cloning and no-superluminal-signalling principles directly imply its impossibility. Wu et al. have presented a more efficient code for this task [4]. Another natural type of summoning task allows any number of calls to be made at call points, requiring that the state be produced at any one of the corresponding return points. Perhaps counter-intuitively, this can be shown to be a strictly harder version of the task [5]. It is possible if and only if the causal diamonds can be ordered in sequence so that the return point of any diamond in the sequence is in the causal future of all call points of earlier diamonds in the sequence. Again, the necessity of this condition follows (with a few extra steps) from the no-superluminal-signalling and no-cloning theorems [5].

The constraints on summoning have cryptographic applications, since they can effectively force Alice to make choices before revealing them to Bob. Perhaps the simplest and most striking of these is a novel type of unconditionally secure relativistic quantum bit commitment protocol, in which Alice sends the unknown state at light speed in one of two directions, depending on her committed bit [6]. The fidelity bounds on approximate quantum cloning imply [6] the sum-binding security condition

(1) $$p_{0}+p_{1}\leq 1+\frac{2}{d+1}\;,$$

where d=dim(H) is the dimension of the Hilbert space of the unknown state and $p_{b}$ is the probability of Alice successfully unveiling bit value *b*.

Summoning is also a natural primitive in distributed quantum computation, in which algorithms may effectively summon a quantum state produced by a subroutine to some computation node that depends on other computed or incoming data.

From a fundamental perspective, the (im)possibility of various summoning tasks may be seen either as results about relativistic quantum theory or as candidate axioms for a reformulation of that theory. They also give a way of exploring and characterising the space of theories generalising relativistic quantum theory. From a cryptographic perspective, we would like to understand precisely which assumptions are necessary for the security of summoning-based protocols. These motivations are particularly strong given the relationship between no-summoning theorems and no-signalling, since we know that quantum key distribution and other protocols can be proven secure based on no-signalling principles alone. In what follows, we characterise that relationship more precisely, and discuss in particular the sense in which summoning-based bit commitment protocols are secure against potentially post-quantum but non-signalling participants. These are participants who may have access to technology that relies on some unknown theory beyond quantum theory. They may thus be able to carry out operations that quantum theory suggests is impossible. However, their technology must not allow them to violate a no-signalling principle. Exactly what this implies depends on which no-signalling principle is invoked. We turn next to discussing the relevant possibilities.

## 2. No-Signalling Principles and No-Cloning

### 2.1. No-Signalling Principles

The relativistic no-superluminal-signalling principle states that no classical or quantum information can be transmitted faster than light speed. We can frame this operationally by considering a general physical system that includes agents at locations $P_{1},\ldots ,P_{n}$. Suppose that the agent at each $P_{i}$ may freely choose inputs labelled by $A_{i}$ and receive outputs $a_{i}$, which may probabilistically depend on their and other inputs. Let $I=\{i_{1},\ldots ,i_{b}\}$ and $J=\{j_{1},\ldots j_{c}\}$ be sets of labels of points such that $P_{i_{k}}⋡P_{j_{l}}$ for all k∈{1,…,b} and l∈{1,…,c}. Then we have

(2) $$\begin{array}{c} P(a_{i_{1}}\ldots a_{i_{b}}|A_{i_{1}}\ldots A_{i_{b}})=\\ p(a_{i_{1}}\ldots a_{i_{b}}|A_{i_{1}}\ldots A_{i_{b}}A_{j_{1}}\ldots A_{j_{c}})\;. \end{array}$$

In other words, outputs are independent of spacelike or future inputs.

The quantum no-signalling principle for an *n*-partite system composed of non-interacting subsystems states that measurement outcomes on any subset of subsystems are independent of measurement choices on the others. If we label the measurement choices on subsystem *i* by $A_{i}$, and the outcomes for this choice by $a_{i}$, then we have

(3) $$P\left(a_{i_{1}}\ldots a_{i_{m}}|A_{i_{1}}\ldots A_{i_{m}}\right)=P\left(a_{i_{1}}\ldots a_{i_{m}}|A_{1}\ldots A_{n}\right)\;.$$

That is, so long as the subsystems are non-interacting, the outputs for any subset are independent of the inputs for the complementary subset, regardless of their respective locations in space-time.

The no-signalling principle for a generalised non-signalling theory extends this to any notional device with localised pairs of inputs (generalising measurement choices) and outputs (generalising outcomes). As in the quantum case, this is supposed to hold true regardless of whether the sites of the localised input/output ports are spacelike separated. Generalized non-signalling theories may include, for example, the hypothetical bipartite Popescu-Rohrlich boxes [7], which maximally violate the CHSH inequality, while still precluding signalling between agents at each site.

### 2.2. The No-Cloning Theorem

The standard derivation of the no-cloning theorem [8,9] assumes a hypothetical quantum cloning device. A quantum cloning device *D* should take two input states, a general quantum state $|\psi \rangle$ and a reference state $|0\rangle$, independent of $|\psi \rangle$. Since *D* follows the laws of quantum theory, it must act linearly. Now we have

(4) $$D|\psi \rangle |0\rangle =|\psi \rangle |\psi \rangle \;,\;D|\psi^{\prime }\rangle |0\rangle =|\psi^{\prime }\rangle |\psi^{\prime }\rangle \;,$$

for a faithful cloning device, for any states $|\psi \rangle$ and $|\psi^{\prime }\rangle$. Suppose that $\langle \psi^{\prime }|\psi \rangle =0$ and that $|\phi \rangle =a|\psi \rangle +b|\psi^{\prime }\rangle$ is normalised. We also have

(5) $$D|\phi \rangle |0\rangle =|\phi \rangle |\phi \rangle \;,$$

which contradicts linearity.

To derive the no-cloning theorem without appealing to linearity, we need to consider quantum theory as embedded within a more general theory that does not necessarily respect linearity. We can then consistently consider a hypothetical post-quantum cloning device *D* which accepts quantum states $|\psi \rangle$ and $|0\rangle$ as inputs and produces two copies of $|\psi \rangle$ as outputs:

(6) $$D|\psi \rangle |0\rangle =|\psi \rangle |\psi \rangle \;.$$

We will suppose that the cloning device functions in this way independent of the history of the input state. We will also suppose that it does not violate any other standard physical principles: in particular, if it is applied at *Q* then it does not act retrocausally to influence the outcomes of measurements at earlier points P≺Q.

We can now extend the cloning device to a bipartite device comprising a maximally entangled quantum state, with a standard quantum measurement device at one end and the cloning device followed by a standard quantum measurement device at the other end. This extended device accepts classical inputs (measurement choices) and produces classical outputs (measurement outcomes) at both ends.

If we now further assume that the joint output probabilities for this extended device, for any set of inputs, are independent of the locations of its components, then we can derive a contradiction with the relativistic no-superluminal signalling principle. First suppose that the two ends are timelike separated, with the cloning device end at point *Q* and the other end at point P≺Q. A complete projective measurement at *P* then produces a pure state at *Q* in any standard version of quantum theory. The cloning device then clones this pure state. Different measurement choices at *P* produce different ensembles of pure states at *Q*. These ensembles correspond to the same mixed state before cloning but to distinguishable mixtures after cloning. The measurement device at *Q* can distinguish these mixtures. Now if we take the first end to be at a point $P^{\prime }$ spacelike separated from *Q*, by hypothesis the output probabilities remain unchanged. This allows measurement choices at $P^{\prime }$ to be distinguished by measurements at *Q* and so gives superluminal signalling [10].

It is important to note that the assumption of location-independence is not logically necessary, nor does it follow from the relativistic no-superluminal-signalling principle alone. Assuming that quantum states collapse in some well defined and localized way as a result of measurements, one can consistently extend relativistic quantum theory to include hypothetical devices that read out a classical description of the local reduced density matrix at any given point, that is the local quantum state that is obtained by taking into account (only) collapses within the past light cone [11]. This means that measurement events at *P*, which we take to induce collapses, are taken into account by the readout device at *Q* if and only if P≺Q. Given such a readout device, one can certainly clone pure quantum states. The device behaves differently, when applied to a subsystem of an entangled system, depending on whether the second subsystem is measured inside or outside the past light cone of the point at which the device is applies. It thus does not satisfy the assumptions of the previous paragraph.

The discussion above also shows that quantum theory augmented by cloning or readout devices is not a generalized non-signalling theory. For consider again a maximally entangled bipartite quantum system with one subsystem at space-time point *P* and the other at a space-like separated point $P^{\prime }$. Suppose that the Hamiltonian is zero and that the subsystem at $P^{\prime }$ will propagate undisturbed to point Q≻P. Suppose that a measurement device may carry out any complete projective measurement at *P* and that at *Q* there is a cloning device followed by another measurement device on the joint (original and cloned) system. As above, different measurement choices at *P* produce different ensembles of pure states at *Q*, which correspond to the same mixed state before cloning but to distinguishable mixtures after cloning. The measurement device at *Q* can distinguish these mixtures. The output (measurement outcome) probabilities at *Q* thus depend on the inputs (measurement choices) at *P*, contradicting Equation (3). Assuming that nature is described by a generalized non-signalling theory thus gives another reason for excluding cloning or readout devices, without assuming that their behaviour is location-independent.

In summary, neither the no-cloning theorem nor cryptographic security proofs based on it can be derived purely from consistency with special relativity. They require further assumptions about the behaviour of post-quantum devices available to participants or adversaries. Although this was noted when cryptography based on the no-signalling principle was first introduced [12], it perhaps deserves re-emphasis.

On the positive side, given these further assumptions, one can prove not only the no-cloning theorem but also quantitative bounds on the optimal fidelities attainable by approximate cloning devices for qubits [10] and qudits [13]. In particular, one can show [13] that any approximate universal cloning device that produces output states $\rho_{0}$ and $\rho_{1}$ given a pure input qudit state $|\psi \rangle$ satisfies the fidelity sum bound

(7) $$\langle \psi |\rho_{0}|\psi \rangle +\langle \psi |\rho_{1}|\psi \rangle \leq 1+\frac{2}{d+1}\;.$$

It is worth stressing that (with the given assumptions) this bound applies for any approximate cloning strategy, with any entangled states allowed as input.

## 3. Summoning-Based Bit Commitments and No-Signalling

We recall now the essential idea of the flying qudit bit commitment protocol presented in Reference [6], in its idealized form. We suppose that space-time is Minkowski and that both parties, the committer (Alice) and the recipient (Bob), have arbitrarily efficient technology, limited only by physical principles. In particular, we assume they both can carry out error-free quantum operations instantaneously and can send classical and quantum information at light speed without errors. They agree in advance on some space-time point *P*, to which they have independent secure access, where the commitment will commence.

We suppose too that Bob can keep a state secure from Alice somewhere in the past of *P* and arrange to transfer it to her at *P*. Alice’s operations on the state can then be kept secure from Bob unless and until she chooses to return information to Bob at some point(s) in the future of *P*. We also suppose that Alice can send any relevant states at light speed in prescribed directions along secure quantum channels, either by ordinary physical transmission or by teleportation.

They also agree on a fixed inertial reference frame and two opposite spatial directions within that frame. For simplicity we neglect the *y* and *z* coordinates and take the speed of light c=1. Let P=(0,0) be the origin in the coordinates (x,t) and the two opposite spatial directions be defined by the vectors $v_{0}=\left(-1,0\right)$ and $v_{1}=\left(1,0\right)$.

Before the commitment begins, Bob generates a random pure qudit $|\psi \rangle \in C^{d}$. This is chosen from the uniform distribution and encoded in some pre-agreed physical system. Again idealizing, we assume the dimensions of this system are negligible and treat it as pointlike. Bob keeps his qudit secure until the point *P*, where he gives it to Alice. To commit to the bit i∈{0,1}, Alice sends the state $|\psi \rangle$ along a secure channel at light speed in the direction $v_{i}$. That is, to commit to 0, she sends the qudit along the line $L_{0}=\left\{\left(-t,t\right),t>0\right\}$; to commit to 1, she sends it along the line $L_{1}=\left\{\left(t,t\right),t>0\right\}$.

For simplicity, we suppose here that Alice directly transmits the state along a secure channel. This allows Alice the possibility of unveiling her commitment at any point along the transmitted light ray. To unveil the committed bit 0, Alice returns $|\psi \rangle$ to Bob at some point $Q_{0}$ on $L_{0}$; to unveil the committed bit 1, Alice returns $|\psi \rangle$ to Bob at some point $Q_{1}$ on $L_{1}$. Bob then tests that the returned qudit is $|\psi \rangle$ by carrying out the projective measurement defined by $P_{\psi }=|\psi \rangle \langle \psi |$ and its complement $\left(I-P_{\psi }\right)$. If he gets the outcome corresponding to $P_{\psi }$, he accepts the commitment as honestly unveiled; if not, he has detected Alice cheating.

Now, given any strategy of Alice’s at *P*, there is an optimal state $\rho_{0}$ she can return to Bob at $Q_{0}$ to maximise the chance of passing his test there, i.e., to maximize the fidelity $\langle \psi |\rho_{0}|\psi \rangle$. There is similarly an optimal state $\rho_{1}$ that she can return at $Q_{1}$, maximizing $\langle \psi |\rho_{1}|\psi \rangle$. The relativistic no-superluminal-signalling principle implies that her ability to return $\rho_{0}$ at $Q_{0}$ cannot depend on whether she chooses to return $\rho_{1}$ at $Q_{1}$ or vice versa. Hence she may return both (although this violates the protocol). The bound (7) on the approximate cloning fidelities implies that

(8) $$\langle \psi |\rho_{0}|\psi \rangle +\langle \psi |\rho_{1}|\psi \rangle \leq 1+\frac{2}{d+1}\;.$$

Since the probability of Alice successfully unveiling the bit value *b* by this strategy is

(9) $$p_{b}=\langle \psi |\rho_{b}|\psi \rangle \;,$$

this gives the sum-binding security condition for the bit commitment protocol

(10) $$p_{0}+p_{1}\leq 1+\frac{2}{d+1}\;.$$

Recall that the bound (7) follows from the relativistic no-superluminal-signalling condition together with the location-independence assumption for a device based on a hypothetical post-quantum cloning device applied to one subsystem of a bipartite entangled state. Alternatively, it follows from assuming that any post-quantum devices operate within a generalized non-signalling theory. The bit commitment security thus also follows from either of these assumptions.

### Security Against Post-Quantum No-Superluminal-Signalling Adversaries?

It is a strong assumption that any post-quantum theory should be a generalized non-signalling theory satisfying Equation (3). So it is natural to ask whether cryptographic security can be maintained with the weaker assumption that other participants or adversaries are able to carry out quantum operations and may also be equipped with post-quantum devices but do not have the power to signal superluminally. It is instructive to understand the limitations of this scenario for protocols between mistrustful parties capable of quantum operations, such as the bit commitment protocol just discussed.

The relevant participant here is Alice, who begins with a quantum state at *P* and may send components along the lightlike lines $PQ_{0}$ and $PQ_{1}$. Without loss of generality we assume these are the only components: she could also send components in other directions but relativistic no-superluminal-signalling means that they cannot then influence her states at $Q_{0}$ or $Q_{1}$.

At any points $X_{0}$ and $X_{1}$ on the lightlike lines, before Alice has applied any post-quantum devices, the approximate cloning fidelity bound again implies that fidelities of the respective components $\rho_{X_{0}}$ and $\rho_{X_{1}}$ satisfy

(11) $$\langle \psi |\rho_{X_{0}}|\psi \rangle +\langle \psi |\rho_{X_{1}}|\psi \rangle \leq 1+\frac{2}{d+1}\;.$$

Now, if Alice possesses a classical no-superluminal-signalling device, such as a Popescu-Rohrlich box, with input and output ports at $X_{0}$ and $X_{1}$ and her agents at these sites input classical information uncorrelated with their quantum states, she does not alter the fidelities $\langle \psi |\rho_{X_{i}}|\psi \rangle$. Any subsequent operation may reduce the fidelities but cannot increase them. More generally, any operation involving the quantum states and devices with purely classical inputs and outputs cannot increase the fidelity sum bound (7). To see this, note that any such operation could be paralleled by local operations within quantum theory if the two states were held at the same point, since hypothetical classical devices with separated pairs of input and output ports are replicable by ordinary probabilistic classical devices when the ports are all at the same site.

We need also to consider the possibility that Alice has no-superluminal signalling devices with quantum inputs and outputs. At first sight these may seem unthreatening. For example, while a device that sends the quantum input from $X_{0}$ to the output at $X_{1}$ and vice versa would certainly make the protocol insecure—Alice could freely swap commitments to 0 and 1—such a device would be signalling.

However, suppose that Alice’s agents each have local state readout devices, which give Alice’s agent at $X_{0}$ a classical description of the density matrix $\rho_{X_{0}}$ and Alice’s agent at $X_{1}$ a classical description of the density matrix $\rho_{X_{1}}$. Suppose also that Alice has carried out an approximate universal cloning at *P*, creating mixed states $\rho_{X_{0}}$ and $\rho_{X_{1}}$ of the form

(12) $$\rho_{X_{i}}=p_{i}|\psi \rangle \langle \psi |+\left(1-p_{i}\right)I\;,$$

where $0<p_{i}<1$. This is possible provided that $p_{0}+p_{1}\leq 1+\frac{2}{d+1}$. From these, by applying their readout devices, each agent can infer $|\psi \rangle$ locally. Alice’s outputs at $X_{i}$ have no dependence on the inputs at $X_{\bar{i}}$. Nonetheless, this hypothetical process would violate the security of the commitment to the maximum extent possible, since it would give $p_{0}+p_{1}=2$.

To ensure post-quantum security, our post-quantum theory thus need assumptions—like those spelled out earlier—that directly preclude state readout devices and other violations of no-cloning bounds.

## 4. Discussion

Classical and quantum relativistic bit commitment protocols have attracted much interest lately, both because of their theoretical interest and because advances in theory [14] and practical implementation [15,16,17] suggest that relativistic cryptography may be in widespread use in the forseeable future.

Much work on these topics is framed in models in which two (or more) provers communicate with one (or more) verifiers, with the provers being unable to communicate with one another during the protocol. Indeed, one round classical relativistic bit commitment protocols give a natural physical setting in which two (or more) separated provers communicate with adjacent verifiers, with the communications timed so that the provers cannot communicate between the commitment and opening phases. The verifiers are also typically unable to communicate but this is less significant given the form of the protocols and the verifiers are sometimes considered as a single entity when the protocol is not explicitly relativistic.

Within the prover-verifier model, it has been shown that no single-round two-prover classical bit commitment protocol can be secure against post-quantum provers who are equipped with generalized no-signalling devices [18]. It is interesting to compare this result with the signalling-based security proof for the protocol discussed above.

First, of course, the flying qudit protocol involves quantum rather than classical communication between “provers” (Alice’s agents) and “verifiers” (Bob’s agents).

Second, as presented, the flying qudit protocol involves three agents for each party. However, a similar secure bit commitment protocol can be defined using just two agents apiece. For example, Alice’s agent at *P* could retain the qudit, while remaining stationary in the given frame, to commit to 0, and send it to Alice’s agent at $Q_{1}$ (as before) to commit to 1. They may unveil by returning the qudit at, respectively, (0,t) or (t,t). In this variant, the commitment is not secure at the point where the qudit is received, but it becomes secure in the causal future of (t/2,t/2).

Third, the original flying qudit protocol illustrates a possibility in relativistic quantum cryptography that is not motivated (and so not normally considered) in standard multi-prover bit commitment protocols. This is that, while there are three provers, communication between them in some directions is possible (and required) during the protocol. Alice’s agent at *P* must be able to send the quantum state to either of the agents at $Q_{0}$ or $Q_{1}$; indeed, a general quantum strategy requires her to send quantum information to both.

Fourth, the security proof of the flying qudit protocol can be extended to generalised no-signalling theories. However, the protocol is not secure if the committer may have post-quantum devices that respect the no-superluminal signalling principle but are otherwise unrestricted. Security proofs require stronger assumptions, such as that the commmitter is restricted to devices allowed by a generalized non-signalling theory.

The same issue arises considering the post-quantum security of quantum key distribution protocols [12], which are secure if a post-quantum eavesdropper is restricted by a generalised no-signalling theory but not if she is only restricted by the no-superluminal-signalling principle. One distinction is that quantum key distribution is a protocol between mutually trusting parties, Alice and Bob, whereas bit commitment protocols involve two mistrustful parties. It is true that quantum key distribution still involves mistrust, in that Alice and Bob mistrust the eavesdropper, Eve. However, if one makes the standard cryptographic assumption that Alice’s and Bob’s laboratories are secure, so that information about operations within them cannot propagate to Eve, one can justify a stronger no-signalling principle [12]. Of course, the strength of this justification may be questioned, given that one is postulating unknown physics that could imply a form of light speed signalling that cannot be blocked. But in any case, the justification is not available when one considers protocols between two mistrustful parties, such as bit commitment, and wants to exclude the possibility that one party (in our case Alice) cannot exploit post-quantum operations within her own laboratories (which may be connected, forming a single extended laboratory).

Our discussion assumed a background Minkowski space-time but generalizes to other space-times with standard causal structure, where the causal relation ≺ is a partial ordering. Neither standard quantum theory nor the usual form of the no-superluminal signalling principle hold in space-times with closed time-like curves, where two distinct points *P* and *Q* may obey both P≺Q and Q≺P. Formulating consistent theories in this context requires further assumptions (see for example Reference [19] for one analysis). The same is true of superpositions of space-times with indefinite causal order [20]. We leave investigation of these cases for future work.
